# Association of Nut Consumption with Dementia and Alzheimer’s Disease

**DOI:** 10.3390/nu18193143

**Published:** 2026-09-24

**Authors:** Ruoling Chen

**Affiliations:** Faculty of Medicine and Health Sciences, University of Wolverhampton, Wolverhampton WV1 1DT, UK; r.chen@wlv.ac.uk; Tel.: +44-(0)1902-328622; Fax: +44-(0)1902-321161

**Keywords:** nut consumption, dementia, case–control study, dietary intakes, older adults

## Abstract

**Background**: Nut consumption reduces risks of cardiovascular disease, respiratory disease and depression. However, its association with dementia and Alzheimer’s disease (AD) remains unclear. **Methods**: A population-based case–control study was conducted in China, including 466 participants aged 52–100 years (233 with dementia). Data on socio-demographic characteristics, lifestyle factors, comorbidities, and dietary intake over the past two years were collected through structured face-to-face interviews. Nut consumption was categorised as non-consumption or <monthly, ≥monthly but <weekly, ≥weekly but <daily, daily, and ≥twice daily. Multiple adjusted logistic regression models were employed to examine the association of nut consumption with dementia and AD. **Results**: Of the 466 participants, 59.0% reported consuming nuts at non-consumption or <monthly, 22.5% monthly, 11.8% weekly, 6.0% daily, and 0.6% ≥ twice daily. Compared with non-consumption or <monthly consumption, the age-adjusted odds ratio (OR) for dementia was 0.31 (95% CI 0.18–0.52) for monthly intake, 0.52 (0.28–0.97) for weekly intake, and 0.32 (0.14–0.72) for daily or higher intake. After further adjustment for gender, education, family income, marital status, smoking, stroke, chronic bronchitis, depression, and other dietary intakes, OR was 0.27 (0.13–0.55) for monthly intake and 0.47 (0.23–0.99) for ≥weekly intake. Among 133 participants with AD, similar associations were observed, with a fully adjusted OR of 0.34 (0.17–0.69) for ≥monthly nut consumption. **Conclusions**: Higher nut consumption was associated with reduced risks of dementia and AD. Increasing nut intake may represent a potential strategy for dementia and AD prevention. However, large-scale cohort and interventional studies are needed to further clarify the association and determine the magnitude of the effect.

## 1. Introduction

Nuts are nutrient-dense, dry, single-seeded fruits enclosed in a hard, woody shell. While strict botanical definitions differ, dietary guidelines and health research generally include tree nuts (almonds, walnuts, cashews) and peanuts (legumes). They are widely consumed as part of human diets and rich in unsaturated fatty acids, plant protein, fibre, vitamins, and bioactive compounds. Dietary intake of nuts has significantly reduced the risks of cardiovascular diseases (CVDs) [1], respiratory diseases, infectious diseases and cancer, and all-cause mortality [2]. Recent research has shown that higher nut consumption was also associated with a lower risk of depression in the population [3]. However, it is unclear whether nut consumption is associated with reduced risks of dementia and Alzheimer’s disease (AD) due to a lack of research.

China has the largest number of people with dementia worldwide and this represents a major and growing public health burden like the rest of the world. It also has the largest global demand for nuts, with annual demand for nuts reaching roughly 4.02 million metric tons, exceeding that of countries such as the United States and India. However, per capita nut consumption in Chinese adults varies, probably due to increased socio-economic disparities in China. In particular, older adults in China have experienced substantial socioeconomic and lifestyle transitions—from early-life deprivation to improved materials conditions in later life [4] following the economic reforms initiated in 1978. This would offer a great opportunity to examine the association of nut consumption with dementia and AD. This study examined data from older adults in a population-based case–control study in China to determine the association of nut consumption with dementia and AD risk.

## 2. Methods

### 2.1. Study Design and Participants

The methods of this study–control study have been described in detail elsewhere [5]. In brief, we conducted the case–control study in Guangzhou, China, in 2020, which was funded by an EU grant from Horizon 2020 MSCA [6], to investigate new risk factors for dementia. Participants were randomly recruited through community health service clinics in Guangzhou city, with eligible age >50 years. Dementia cases were identified via the dementia management system and enrolled from Huiai Hospital in Guangzhou, with diagnoses based on ICD-10 criteria. Dementia subtypes included Alzheimer’s disease, vascular dementia, and others. Controls were selected from older adults attending annual health checks and were screened to exclude dementia using standard cognitive assessments. A total of 233 cases and 233 controls were identified for the study. Their dementia and non-dementia diagnoses were further confirmed by a neurologist. All dementia patients in the study had been diagnosed within the past year and were ambulatory during interviews.

Informed consent was obtained from all participants. In participants where an individual was unable to provide informed consent due to disability or limited educational level, their next of kin or caregivers would provide assent for the interview. Face-to-face interviews were conducted in safe settings within Huiai Hospital or other designated places including participants’ homes. Participants were encouraged to have a family member or caregiver present during the interview for support. The main interview material was a validated Chinese General Health and Risk Factor Questionnaire [7], which collected information on sociodemographic characteristics, lifestyle factors, medical history, and dietary intake [5,7]. Nut consumption over the past two years was assessed in five frequency categories: (i) non-consumption or <once a month (i.e., <monthly); (ii) ≥once per month but <once a week (i.e., monthly); (iii) ≥once per week but <once a day (i.e., weekly); (iv) ≥once per day but <twice a day (i.e., daily); and (v) ≥twice a day (i.e., >daily).

### 2.2. Statistical Analysis

Participant characteristics across different levels of nut consumption were summarised using means (standard deviations) for continuous variables and percentages for categorical variables. Differences among category groups of nut consumption were assessed using one-way analysis of variance (ANOVA) for continuous variables and the χ^2^ test for categorical variables. Differences in other dietary intakes across nut consumption categories were examined using the Kruskal–Wallis test. The association between nut consumption and dementia was assessed using binary logistic regression. Odds ratios (ORs) and 95% confidence intervals (CIs) were estimated for dementia across categories of nut consumption, with the group of non-consumption or <once a month nut consumption as the reference. Three models were constructed: model 1 adjusted for age, gender, and sociodemographic status; model 2 additionally adjusted for lifestyle variables and comorbidities; and model 3 further adjusted for other dietary intakes. Missing data in covariates were treated as a separate category in the analysis. Subgroup analyses were conducted for Alzheimer’s disease. All analyses were performed using IBM SPSS Statistics version 28 (IBM Co., Armonk, NY, USA).

## 3. Results

Among the 466 participants, 275 (59.0%) documented non-consumption of nuts or consumption <once a month, 105 (22.5%) declared monthly consumption, 55 (11.8%) weekly, 28 (6.0%) daily, and 3 (0.6%) ≥twice a day. Owing to the small number of participants in group with nut consumption ≥twice a day, this group was combined with the daily category for subsequent analyses.

Table 1 shows the characteristics of participants according to levels of nut consumption. Compared with participants in the non-consuming or <once a month category, those with overall nut intakes were more likely to be younger, have higher levels of education and family income, be married, and have lower levels of stroke, chronic bronchitis (overall 5.6% vs. 10.5%) and depression (2.7% vs. 5.5%). No significant differences were observed across nut consumption categories for other factors presented in Table 1.

Table 2 displays other dietary intakes across categories of nut consumption. Kruskal–Wallis tests indicated significant differences in the intake of egg, beef, lamb, poultry, fish and fruits across nut consumption categories, with higher intakes of these foods observed among participants with greater nut consumption. No significant differences were found in the intake of pork or vegetables across nut consumption categories.

Table 3 presents the distribution and odds ratios of dementia across categories of nut consumption. The pattern of nut consumption between participants with and without dementia was significantly different; there was a larger percentage of those not eating nuts or eating them <monthly and a smaller percentage of ≥daily, weekly and monthly nut consumption in participants with dementia than those without dementia. Age-adjusted OR of dementia significantly decreased with the nut consumption at monthly (0.31, 95%CI 0.18–0.52), weekly (0.52, 0.28–0.97) and ≥daily rates (0.32, 0.14–0.72) versus those not consuming nuts or consuming them <monthly (Table 3).

In the multiple adjusted analyses (Table 4), which combined the groups of weekly and ≥daily nut consumption for statistical power, we found that the reduced ORs in those with higher levels of nut consumption remained significant. In Model 1 with adjustment for age, gender, education and family income, the OR of dementia in those with monthly nut consumption was 0.35 (0.20–0.60), and in those with ≥weekly consumption it was 0.49 (0.28–0.85), versus those not consuming nuts or consuming them <monthly. Further adjustment for marital status, smoking, and comorbidities (Model 2) slightly attenuated these associations (Table 4). Additional adjustments for other dietary intakes (Model 3) did not change the significant association of nut consumption with reduced odds of dementia.

Of the 233 participants with dementia, 133 were diagnosed as having Alzheimer’s disease. Table 5 shows the number, % and odds ratios of AD across categories of nut consumption. The distribution of nut consumption significantly differed between participants with and without AD, presenting a higher proportion of low consumption (non-consumption or <once per month) and lower proportions of higher consumption categories among those with AD versus the controls. Age-adjusted logistic regression analysis demonstrated a significant inverse association between nut consumption and Alzheimer’s disease; the OR was 0.34 (0.17–0.65) for monthly consumption and 0.53 (0.28–0.98) for ≥weekly consumption compared with those with non-consumption or <once a month consumption. The multivariate adjusted analysis for AD cases, which combined the groups of nut consumption at monthly and ≥weekly rates for maintaining statistical power, showed that the association of nut consumption with AD was also significant. The fully adjusted OR for Alzheimer’s disease with ≥monthly of nuts versus non-consumption or <monthly was 0.45 (0.27–0.75; *p* = 0.002) in Model 1, 0.51 (0.29–0.92; *p* = 0.024) in Model 2 and 0.34 (0.17–0.69; *p* = 0.003) in Model 3. These were comparable with the ORs observed for all types of dementia (the corresponding ORs for dementia were 0.41 (0.27–0.63; *p* < 0.001) in model 1, 0.49 (0.30–0.80; *p* = 0.004) in model 2, and 0.35 (0.19–0.63; *p* < 0.001) in model 3).

## 4. Discussion

This population-based study in China found that a reduced risk of dementia was related to increased consumption of nuts on a monthly, weekly and daily basis compared with non-consumption or <once a month consumption. Such an inverse association was independent of important co-variates, such as age, gender, education, family income, smoking, co-morbidities and other dietary intakes (e.g., egg, fish). Similar findings were observed for Alzheimer’s disease; compared to those with non-consumption or <once a monthly consumption of nuts, the participants with ≥monthly consumption of nuts had significantly reduced AD risk.

Despite the current literature lacking research on nut consumption associated with dementia and AD, several studies have examined its association with cognitive impairment in older adults [8,9,10,11]. In the USA, Samieri et al. [8] analysed the data of 6174 women aged ≥65 years in the Women’s Health Study. The participants provided dietary information in 1998 and completed a cognitive battery 5 years later, followed by two assessments at 2-year intervals. The authors did not find that the quintile of total nut consumption was significantly associated with global cognitive function and verbal memory. In the Netherlands, Nooyens et al. [9] examined the data of 2613 men and women aged 43–70 years from the Doetinchem Cohort Study of 1995–2002. Multivariate linear regression analyses for baseline cross-sectional data showed modest positive associations between nut intake and multiple cognitive domains, but these associations were attenuated and no longer significant in longitudinal analysis. In contrast, findings from studies in China and Spain suggest potential benefits. Li et al. [10] analysed the data from the Zhejiang Ageing and Health Cohort Study including 9028 participants aged ≥60 years in China. Nut consumption was evaluated in a baseline questionnaire beginning in 2014. Cognitive function was assessed repeatedly through the Mini-Mental State Examination (MMSE) at baseline and three waves of follow-up (2015, 2016, and 2019–2020). The authors found that compared with non-consumers or <weekly consumers, participants consuming ≥70 g/week of nuts had 17% lower risks of cognitive impairment during the 6-year follow-up (RR 0.83, 95% CI 0.75–0.91), whereas no association was found among those consuming <70 g/week of nuts. In Spain, Ni et al. [11] examined the data of 6630 participants aged 55–75 years with overweight/obesity and metabolic syndrome from the Predimed-Plus clinical trial. The participants completed a validated semi-quantitative food frequency questionnaire and a comprehensive battery of neuropsychological tests at baseline and a 2 y follow-up, including assessments for global, general, attention, and executive function domains. The cohort data analysis showed that compared with participants consuming <1 serving/week of nuts, those categorised as consuming ≥3–<7 and ≥7 servings/week demonstrated more favourable changes in general cognitive performance. A recent systematic literature review [12] suggested that beneficial associations of nut consumption with cognitive impairment were more likely to be observed in populations at higher risk of cognitive decline.

Few studies have been conducted to examine the association between nut consumption and dementia risk. In our systematic literature search (PROSPERO Register Nos. CRD420261391069), only one study [13] was found on the topic. Bizzozero-Peroni et al. [13] analysed the UK Biobank data from 50,386 participants aged 37–73 years, with baseline information collected between 2007 and 2012 and follow-up through 2023. Nut consumption was assessed at baseline using the Oxford WebQ 24 h dietary questionnaire, and dementia and its different types were identified through self-reported medical diagnosis, hospital records and death registries. Multiple adjusted hazard ratio (HR) for incident dementia among those with daily nut consumption (>0–3 or more handfuls) compared with no consumption was 0.88 (95% CI 0.77–0.99). However, the association with Alzheimer’s disease was not statistically significant (HR 0.89, 95% CI 0.73–1.08). Our study in China revealed a significant association of nut consumption with reduced risks of dementia and AD. The magnitude of the association in our China study appears greater than that reported in UK populations [13]. It may reflect differences in life course exposures and population characteristics [4].

The consumption of nuts has increased in recent years due to their high nutritional value and their association with numerous health benefits [14]. Many older adults in China incorporate nuts into their daily diets, owing to their high nutritional value and links to several health benefits. Nuts are commonly consumed as snacks or included in meals such as breakfast dishes, salads, and baked goods, contributing to overall nutrient intake. Nuts are also frequently included in flexitarian dietary patterns, where moderate consumption can help meet protein requirements. In recent years, growing evidence has assessed the role of nut consumption in supporting brain health across the life course [10,11,12]. Tree nuts (such as walnuts, almonds, Brazil nuts, cashews, hazelnuts, and pistachios) and peanuts are energy-dense foods with a favourable nutrient profile [15]. They are rich in essential nutrients and bioactive compounds such as mono- and polyunsaturated fatty acids, low-sodium minerals, polyphenols, and phytosterols, which may act synergistically to produce a wide range of neurocognitive health benefits [16,17,18]. Bioavailable phytochemicals in nuts have demonstrated anti-inflammatory and antioxidant properties, which could play a beneficial role in delaying the onset or progression of cognitive impairment [19].

Nut consumption could reduce dementia risk through several biological and behavioural pathways. Nuts have a distinctive nutrition profile, characterised by high levels of unsaturated fatty acids, polyunsaturated fatty acids (PUFAs), phytochemicals, and micronutrients. These components could improve endothelial function [20], glucose regulation and fasting glucose concentrations [21], and maintenance of neuronal membrane integrity [22], and impart anti-inflammatory and vascular-protective effects [23]. The antioxidant and anti-inflammatory properties of nut-derived bioactive compounds may help reduce oxidative stress and vascular dysfunction [24,25]. Previous population-based studies have shown that nut consumption was also linked to lower risks of metabolic syndrome [26] and CVD [1,27]; for example, consuming a handful of nuts per day induced a 19% lower risk of CVD incidence in the general population [1]. Furthermore, nut intake has been associated with a lower risk of depression [3], while depression itself is a recognised risk factor for dementia [28]. Beyond these direct effects, incorporating nuts into the diet may improve overall dietary quality [29,30]. For example, walnut consumption has been shown to displace less healthy sources and is associated with lower intake of total and refined carbohydrates, animal protein, saturated fatty acids, and sodium, as well as higher intake of PUFAs and plant-based proteins [31,32]. In this study population, higher nut consumption was also positively associated with greater intake of foods such as eggs and fish, which have been linked to reduced risk of dementia [5,7]. Taken together, these benefits of nut intake could contribute to lowering the risk of dementia and AD in the population.

Peanuts are the most consumed nut among older adults in China [33,34]. Their popularity is likely driven by affordability, availability, and versatility, as they are widely used both as a staple ingredient and as a snack. Although botanically classified as legumes, peanuts have a nutrient profile comparable to that of tree nuts and exhibit similar health effects [27]. Evidence suggests that peanut consumption may support cognitive function, including memory, in the general population [35,36]. Walnuts are the second most consumed nuts in China [33,34], the world’s largest producer and consumer of walnuts. In Chinese culture, walnuts are traditionally believed by many people to benefit brain health, partly due to their resemblance to the human brain. Emerging evidence supports this perception, with studies indicating that walnut consumption may improve cognitive performance, potentially owning to their high contents of α-linolenic acid, a plant-based n–3 fatty acid [31,37,38]. In addition, walnuts, peanuts and other nuts are rich sources of dietary fibre, B vitamins, minerals, and antioxidant compounds [24,27], which may help protect against oxidative damage and support cognitive function. Thus, it is not surprising that our population-based study in China identified a significant association of nut consumption with a lower risk of dementia and AD.

## 5. Strengths and Limitations of This Study

To the best of my knowledge, this study is the first to examine the association of nut consumption with dementia in China. The findings of this study provide novel evidence suggesting that higher nut intake would be associated with a lower risk of dementia and AD. The data analysis included a range of important confounders for adjustment, such as age, educational level, family income, stroke, depression and other dietary intakes such as red meat, poultry meat, egg and fish, thereby minimising residual effects. The study has limitations. First, this study had a case–control design, limiting inference on causal direction. However, it is a well-designed and meticulously conducted population study [5]; participants’ family members supported them in answering the questions in the interview where needed, which reduced recall bias. The baseline measuring the frequency of nut consumption over the past two years could help contribute to causal inference. Our data on the association between higher consumption of nuts and low risk of stroke, chronic respiratory diseases and depression (Table 1) were consistent with those in previous studies [1,2,3]. Furthermore, the inverse association we observed in this study was consistent with those in longitudinal studies in the UK [13] and in China [10,39]. Second, nut intake was assessed only by consumption frequency rather than quantity or type of nuts, limiting assessment of dose–response relationships and introducing exposure misclassification. It may have potentially attenuated the observed association, and our findings may be more conservative. Third, although the study analysis adjusted for a number of confounders, residual confounding effects may remain likely. We do not have data on biomarkers such as lipid profiles, omega-3 fatty acids, B vitamins, lutein, and inflammatory markers, or neuroimaging measures for adjustments in logistic regression models. Higher nut consumption may be a marker of an overall healthier lifestyle, greater health consciousness, or higher-quality dietary patterns. In the full adjustment analysis (model 3), however, we adjusted for educational level, family income, smoking, co-morbidities and dietary intake (including red meat, egg, fish and fruits, which involved lipid profiles, omega-3 fatty acids, B vitamins and, lutein). Thus, our data analysis has minimised the residual confounding effect. Fourth, the numbers of participants across the nut intake categories are not similar and vary significantly, which may reflect the real-word situation of nut consumption in older adults in China, but lowers the statistical power of the data analysis. A large prospective study in China is needed to further clarify the association of nut consumption with dementia and AD, including its effect size and dose–response relationship.

## 6. Conclusions and Implications

Regular consumption of nuts is significantly associated with reduced risks of dementia and AD in Chinese older adults. The findings may have implications for public health strategies aimed at reducing the burden of dementia globally. It is especially important for dementia prevention under the current circumstances, with no effective curative treatments and huge costs. Promoting moderate nut consumption as part of a balanced diet could represent a feasible and scalable approach to support cognitive health among older adults. Interventional studies and randomised controlled trials (RCTs) should be undertaken to confirm the effect of nut consumption on dementia and AD in older adults, supporting a potential preventive strategy for dementia and Alzheimer’s disease in later life.

## Figures and Tables

**Table 1:** Characteristics of participants in Guangzhou case–control study, China.

	Consumption of Nuts in the Past Two Years								
Variable ^†^	Non-Consuming or <Monthly		Monthly		Weekly		≥Daily		*p* ^†^
	n = 275, (%)		n = 105, (%)		n = 55, (%)		n = 31, (%)		
**Basic characteristics **									
*Age (years)*									
Mean (SD)	74.7	10.0	70.1	7.7	73.2	9.0	76.1	8.2	<0.001
*Gender*									
Women	177	64.4	67	63.8	33	60.0	19	61.3	0.930
Men	98	35.6	38	36.2	22	40.0	12	38.7	
*Educational years*									
>Median (6 yrs)	117	42.5	58	55.2	37	67.3	15	48.4	<0.001 *^&^*
≤Median	158	57.5	47	44.8	18	32.7	16	51.6	
*Family income per year (RMB)*									
≥70 k	38	13.8	12	11.4	11	20.0	9	29.0	<0.001
50k–<70k	45	16.4	19	18.1	6	10.9	2	6.5	
30k–<-50k	77	28.0	52	49.5	27	49.1	7	22.6	
<30k	115	41.8	22	21.0	11	20.0	13	41.9	
*Marriage*									
Married or cohabiting	163	59.3	78	74.3	38	69.1	21	67.7	0.010
Never married, separated, and divorced	17	6.2	6	5.7	8	14.5	1	3.2	
Widow	93	33.8	21	20.0	9	16.4	9	29.0	
Missing	2	0.7	0		0				
*Smoking*									
No	220	80.0	90	85.7	46	83.6	24	77.4	0.729 ^‡^
Occasionally/formally	33	12.0	7	6.7	5	9.1	3	9.7	
Regularly	22	8.0	8	7.6	4	7.3	4	12.9	
*Drinking alcohol (wine) over the past 2 years*									
Non-consuming or less than once per month	268	97.5	101	96.2	54	98.2	28	90.3	0.165 ^‡^
More than once a month	7	2.5	4	3.8	1	1.8	3	9.7	
**Cardiovascular disease and risk factors**									
*Hypertension status*									
No	129	46.9	53	50.5	26	47.3	11	35.5	0.539
Yes	146	53.1	52	49.5	29	52.7	20	64.5	
*High blood cholesterol*									
No	238	86.5	90	85.7	49	89.1	23	74.2	0.170
Yes	32	11.6	14	13.3	6	10.9	8	25.8	
Missing	5	1.8	1	1.0	0		0		
*Diabetes*									
No	224	81.5	83	79.0	41	74.5	26	83.9	0.629
Yes	51	18.5	22	21.0	14	25.5	5	16.1	
*Coronary heart disease*									
No	250	90.9	93	88.6	50	90.9	29	93.5	0.810
Yes	24	8.7	12	11.4	5	9.1	2	6.5	
Missing	1	0.4	0		0		0		
*Stroke*									
No	225	81.8	99	94.3	53	96.4	27	87.1	0.001
Yes	48	17.5	6	5.7	1	1.8	4	12.9	
Missing	2	0.7	0		1	1.8	0		
*Kidney disease*									
No	261	94.9	104	99.0	54	98.2	29	93.5	0.141 ^‡^
Yes	14	5.1	1	1.0	1	1.8	2	6.5	
*Head hurt*									
No	256	93.1	100	95.2	53	96.4	28	90.3	0.612 ^‡^
Yes	19	6.9	5	4.8	2	3.6	3	9.7	
*Chronic bronchitis*									
No	235	85.5	102	97.1	50	90.9	25	80.6	0.001 ^‡^
Yes	29	10.5	1	1.0	4	7.3	5	16.1	
Missing	11	4.0	2	1.9	1	1.8	1	3.2	
*Depression*									
No	249	90.5	101	96.2	54	98.2	27	87.1	0.050 ^‡^
Yes	15	5.5	2	1.9	0	0.0	3	9.7	
Missing	11	4.0	2	1.9	1	1.8	1	3.2	
*Migraine*									
No	242	88.0	97	92.4	52	94.5	26	83.9	0.271
Yes	32	11.6	8	7.6	3	5.5	5	16.1	
Missing	1	0.4	0		0		0		
*Parkinson’s disease*									
No	231	84.0	102	97.1	49	89.1	28	90.3	0.295 ^‡^
Yes	17	6.2	3	2.9	4	7.3	3	9.7	
Unknown/refused-answer	27	9.8	0		2	3.6	0		

^†^ *p*-values in the chi-square test are calculated based on available data, not including missing data if any. ^‡^ With Fisher–Freeman–Halton Exact Test. *^&^* Non-parametric Kruskal–Wallis H test.

**Table 2:** Dietary intake among participants in Guangzhou case–control study, China.

Variable	Consumption of Eggs in the Past Two Years								
Dietary Intake in the Past Two Years	Non-Consuming or <Monthly		Monthly		Weekly		≥Daily		*p ^&^*
Freq Level	n		n		n		n		
*Consumption of Pork*									
>median	122	44.4	35	33.3	22	40.0	14	45.2	0.709
≤median (3.0)	153	55.6	70	66.7	33	60.0	17	54.8	
*Consumption of Beef*									
>median	79	28.7	62	59.0	33	60.0	11	35.5	<0.001
≤median (1.0)	196	71.3	43	41.0	22	40.0	20	64.5	
*Consumption of Lamb*									
>median	37	13.5	40	38.1	22	40.0	6	19.4	<0.01
≤median (1.0)	238	86.5	65	61.9	33	60.0	25	80.6	
*Consumption of Poultry Meat*									
>median	48	17.5	16	15.2	13	23.6	12	38.7	0.019
≤median (3.0)	227	82.5	89	84.8	42	76.4	19	61.3	
*Consumption of Egg*									
>median	94	34.2	38	36.2	27	49.1	17	54.8	0.012
≤median (3.0)	181	65.8	67	63.8	28	50.9	14	45.2	
*Consumption of Fish*									
>median	71	25.8	26	24.8	16	29.1	15	48.4	0.005
≤median (3.0)	204	74.2	79	75.2	39	70.9	16	51.6	
*Consumption of Vegetables*									
>median	111	40.4	46	43.8	26	47.3	7	22.6	0.310
≤median (4.0)	164	59.6	59	56.2	29	52.7	24	77.4	
*Consumption of Fruits*									
>median	112	40.7	45	45	37	67.3	29	93.5	<0.001
≤median (3.0)	163	59.3	60	60	18	32.7	2	6.5	

*^&^* Non-parametric Kruskal–Wallis H test.

**Table 3:** Number, % and age-adjusted OR of cases at different levels of nut consumption among participants in Guangzhou case–control study, China.

Nut Consumption	All Participants		With Dementia		Without Dementia			Age-Adjusted Analysis		
In the Past 2 Years	N = 466	%	N = 233	%	N = 233	%	*p*	OR	95% CI	*p*
Non-consuming or <Monthly	275	59.0	168	72.1	107	45.9	<0.001	1.00		
Monthly	105	22.5	29	12.4	76	32.6		0.31	0.18–0.52	<0.001
Weekly	55	11.8	24	10.3	31	13.3		0.52	0.28–0.97	0.038
≥Daily	31	6.7	12	5.2	19	8.2		0.32	0.14–0.72	0.006

**Table 4:** Multiple adjusted OR of dementia at different levels of nut consumption among participants in Guangzhou case–control study, China.

Nut Consumption	Model 1			Model 2			Model 3		
In the Past 2 Years	OR^1^	95% CI	*p*	OR^2^	95% CI	*p*	OR^3^	95% CI	*p*
Non-consuming or <Monthly	1.00			1.00			1.00		
Monthly	0.35	0.20–0.60	<0.001	0.45	0.25–0.82	0.009	0.27	0.13–0.55	<0.001
≥Weekly	0.49	0.28–0.85	0.011	0.54	0.29–1.00	0.050	0.47	0.23–0.99	0.046

OR^1^ in Model 1: adjusted for age (cont.), gender, education and family income; OR^2^ in Model 2: adjusted for those in Model 1, plus marital status, smoking, stroke, chronic bronchitis and depression; OR^3^ in Model 3: adjusted for those in Model 2, plus consumption of beef, lamb, poultry meat, egg, fish and fruit.

**Table 5:** Number, % and age-adjusted OR of Alzheimer’s disease at different levels of nut consumption among participants in Guangzhou case–control study, China.

Nut Consumption	With AD		Without AD/Dementia			Age-Adjusted Analysis		
In the Past 2 Years	N = 133	%	N = 233	%	*p*	OR	95% CI	*p*
Non-consuming or <Monthly	96	72.2	107	45.9	<0.001	1.00		
Monthly	15	11.3	76	32.6		0.34	0.17–0.65	0.001
≥Weekly	22	16.5	50	21.5		0.53	0.28–0.98	0.044

## Data Availability

The original contributions presented in the study are included in the article, further inquiries can be directed to the corresponding author.

## References

[1] Glenn A.J., Aune D., Freisling H., Mohammadifard N., Kendall C.W.C., Salas-Salvado J., Jenkins D.J.A., Hu F.B., Sievenpiper J.L. (2023). Nuts and Cardiovascular Disease Outcomes: A Review of the Evidence and Future Directions. Nutrients.

[2] Balakrishna R., Bjornerud T., Bemanian M., Aune D., Fadnes L.T. (2022). Consumption of Nuts and Seeds and Health Outcomes Including Cardiovascular Disease, Diabetes and Metabolic Disease, Cancer, and Mortality: An Umbrella Review. Adv. Nutr..

[3] Fernandez-Rodriguez R., Jimenez-Lopez E., Garrido-Miguel M., Martinez-Ortega I.A., Martinez-Vizcaino V., Mesas A.E. (2022). Does the evidence support a relationship between higher levels of nut consumption, lower risk of depression, and better mood state in the general population? A systematic review. Nutr. Rev..

[4] Chen R., Wei L., Hu Z., Qin X., Copeland J.R., Hemingway H. (2005). Depression in older people in rural China. Arch. Intern. Med..

[5] Igbinigie P.O., Chen R., Tang J., Dregan A., Yin J., Acharya D., Nadim R., Chen A., Bai Z., Amirabdollahian F. (2024). Association between Egg Consumption and Dementia in Chinese Adults. Nutrients.

[6] Tang J., Chen A., He F., Shipley M., Nevill A., Coe H., Hu Z., Zhang T., Kan H., Brunner E. (2023). Association of air pollution with dementia: A systematic review with meta-analysis including new cohort data from China. Environ. Res..

[7] Bakre A.T., Chen R., Khutan R., Wei L., Smith T., Qin G., Danat I.M., Zhou W., Schofield P., Clifford A. (2018). Association between fish consumption and risk of dementia: A new study from China and a systematic literature review and meta-analysis. Public Health Nutr..

[8] Samieri C., Grodstein F., Rosner B.A., Kang J.H., Cook N.R., Manson J.E., Buring J.E., Willett W.C., Okereke O.I. (2013). Mediterranean diet and cognitive function in older age. Epidemiology.

[9] Nooyens A.C.J., Bueno-de-Mesquita H.B., van Boxtel M.P.J., van Gelder B.M., Verhagen H., Verschuren W.M.M. (2011). Fruit and vegetable intake and cognitive decline in middle-aged men and women: The Doetinchem Cohort Study. Br. J. Nutr..

[10] Li F., Jiang W., Wang J., Zhang T., Gu X., Zhai Y., Wu M., Xu L., Lin J. (2022). Beneficial Effects of Nut Consumption on Cognitive Function Among Elderly: Findings From a 6-Year Cohort Study. Front. Aging Neurosci..

[11] Ni J., Nishi S.K., Babio N., Ros E., Basterra-Gortari F.J., Corella D., Castañer O., Martínez J.A., Alonso-Gómez Á.M., Wärnberg J. (2023). Higher versus lower nut consumption and changes in cognitive performance over two years in a population at risk of cognitive decline: A cohort study. Am. J. Clin. Nutr..

[12] Theodore L.E., Kellow N.J., McNeil E.A., Close E.O., Coad E.G., Cardoso B.R. (2021). Nut Consumption for Cognitive Performance: A Systematic Review. Adv. Nutr..

[13] Bizzozero-Peroni B., Diaz-Goni V., Beneit N., Oliveira A., Jimenez-Lopez E., Martinez-Vizcaino V., Mesas A.E. (2025). Nut consumption is associated with a lower risk of all-cause dementia in adults: A community-based cohort study from the UK Biobank. Geroscience.

[14] Martini D., Godos J., Marventano S., Tieri M., Ghelfi F., Titta L., Lafranconi A., Trigueiro H., Gambera A., Alonzo E. (2021). Nut and legume consumption and human health: An umbrella review of observational studies. Int. J. Food Sci. Nutr..

[15] George E.S., Daly R.M., Tey S.L., Brown R., Wong T.H.T., Tan S. (2022). Perspective: Is it Time to Expand Research on “Nuts” to Include “Seeds”? Justifications and Key Considerations. Adv. Nutr..

[16] Ros E., Singh A., O’Keefe J.H. (2021). Nuts: Natural Pleiotropic Nutraceuticals. Nutrients.

[17] Nijssen K.M., Mensink R.P., Plat J., Ivanov D., Preissl H., Joris P.J. (2024). Mixed nut consumption improves brain insulin sensitivity: A randomized, single-blinded, controlled, crossover trial in older adults with overweight or obesity. Am. J. Clin. Nutr..

[18] Nijssen K.M.R., Mensink R.P., Plat J., Joris P.J. (2023). Longer-term mixed nut consumption improves brain vascular function and memory: A randomized, controlled crossover trial in older adults. Clin. Nutr..

[19] Gorji N., Moeini R., Memariani Z. (2018). Almond, hazelnut and walnut, three nuts for neuroprotection in Alzheimer’s disease: A neuropharmacological review of their bioactive constituents. Pharmacol. Res..

[20] Neale E.P., Tapsell L.C., Guan V., Batterham M.J. (2017). The effect of nut consumption on markers of inflammation and endothelial function: A systematic review and meta-analysis of randomised controlled trials. BMJ Open.

[21] Kim Y., Keogh J.B., Clifton P.M. (2019). Does Nut Consumption Reduce Mortality and/or Risk of Cardiometabolic Disease? An Updated Review Based on Meta-Analyses. Int. J. Environ. Res. Public Health.

[22] Cutuli D. (2017). Functional and Structural Benefits Induced by Omega-3 Polyunsaturated Fatty Acids During Aging. Curr. Neuropharmacol..

[23] Ren J., Chung S.H. (2007). Anti-inflammatory effect of alpha-linolenic acid and its mode of action through the inhibition of nitric oxide production and inducible nitric oxide synthase gene expression via NF-kappaB and mitogen-activated protein kinase pathways. J. Agric. Food Chem..

[24] Bolling B.W., Chen C.O., McKay D.L., Blumberg J.B. (2011). Tree nut phytochemicals: Composition, antioxidant capacity, bioactivity, impact factors. A systematic review of almonds, Brazils, cashews, hazelnuts, macadamias, pecans, pine nuts, pistachios and walnuts. Nutr. Res. Rev..

[25] Kim Y., Keogh J.B., Clifton P.M. (2017). Benefits of Nut Consumption on Insulin Resistance and Cardiovascular Risk Factors: Multiple Potential Mechanisms of Actions. Nutrients.

[26] Zhang Y., Zhang D. (2019). Relationship Between Nut Consumption and Metabolic Syndrome: A Meta-Analysis of Observational Studies. J. Am. Coll. Nutr..

[27] Ros E., Tapsell L.C., Sabate J. (2010). Nuts and berries for heart health. Curr. Atheroscler. Rep..

[28] Chen R., Hu Z., Wei L., Qin X., McCracken C., Copeland J.R. (2008). Severity of depression and risk for subsequent dementia: Cohort studies in China and the UK. Br. J. Psychiatry.

[29] Fraser G.E., Bennett H.W., Jaceldo K.B., Sabate J. (2002). Effect on body weight of a free 76 Kilojoule (320 calorie) daily supplement of almonds for six months. J. Am. Coll. Nutr..

[30] Jaceldo-Siegl K., Sabate J., Rajaram S., Fraser G.E. (2004). Long-term almond supplementation without advice on food replacement induces favourable nutrient modifications to the habitual diets of free-living individuals. Br. J. Nutr..

[31] Sala-Vila A., Valls-Pedret C., Rajaram S., Coll-Padros N., Cofan M., Serra-Mir M., Pérez-Heras A.M., Roth I., Freitas-Simoes T.M., Doménech M. (2020). Effect of a 2-year diet intervention with walnuts on cognitive decline. The Walnuts And Healthy Aging (WAHA) study: A randomized controlled trial. Am. J. Clin. Nutr..

[32] Bitok E., Jaceldo-Siegl K., Rajaram S., Serra-Mir M., Roth I., Feitas-Simoes T., Ros E., Sabaté J. (2017). Favourable nutrient intake and displacement with long-term walnut supplementation among elderly: Results of a randomised trial. Br. J. Nutr..

[33] Sun Y., Jiang C.Q., Cheng K.K., Zhang W.S., Leung G.M., Lam T.H., Schooling C.M. (2015). Nut Consumption and Cardiovascular Risk in Older Chinese: The Guangzhou Biobank Cohort Study. PLoS ONE.

[34] Li M., Shi Z. (2019). A Prospective Association of Nut Consumption with Cognitive Function in Chinese Adults aged 55+—China Health and Nutrition Survey. J. Nutr. Health Aging.

[35] Kerkhof L., Mensink R.P., Plat J., Nijssen K.M.R., Joris P.J. (2025). Longer-term skin-roasted peanut consumption improves brain vascular function and memory: A randomized, single-blind, controlled crossover trial in healthy older adults. Clin. Nutr..

[36] Parilli-Moser I., Dominguez-Lopez I., Trius-Soler M., Castellvi M., Bosch B., Castro-Barquero S., Estruch R., Hurtado-Barroso S., Lamuela-Raventós R.M. (2021). Consumption of peanut products improves memory and stress response in healthy adults from the ARISTOTLE study: A 6-month randomized controlled trial. Clin. Nutr..

[37] Chauhan A., Chauhan V. (2020). Beneficial Effects of Walnuts on Cognition and Brain Health. Nutrients.

[38] Bell L., Dodd G.F., Jeavons M., Fisher D.R., Whyte A.R., Shukitt-Hale B., Williams C.M. (2025). The impact of a walnut-rich breakfast on cognitive performance and brain activity throughout the day in healthy young adults: A crossover intervention trial. Food Funct..

[39] He F., Tang J., Zhang T., Lin J., Li F., Gu X., Chen A., Nevill A., Chen R. (2021). Impact of air pollution exposure on the risk of Alzheimer’s disease in China: A community-based cohort study. Environ. Res..

